# Diaphragmatic Hernia

**Published:** 1841-07

**Authors:** George W. Bayless

**Affiliations:** Demonstrator of Anatomy in the Medical Institute of Louisville


ART. II.—*Diaphragmatic Hernia.* By GEORGE W. BAYLESS, M. D., Demonstrator of Anatomy in the Medical Institute of Louisville.*

LOUISVILLE, Wednesday, June 2, 1841.

The committee on Post-mortem Examinations, Morbid Anatomy, and Pathology, beg leave to make this their first report; being an account of a case of Diaphragmatic Hernia, together with some remarks on that disease.

### REPORT.

As one of the attending physicians of the Louisville Marine and Charity Hospital, a member of your committee was notified to attend a post-mortem examination of the body of John ——, on the 19th of September, 1840; and, upon dissection, there were found some morbid appearances which are deemed of sufficient interest to form the subject of a report to the Association. But before giving an account of them, we will briefly enumerate such facts in the history of the individual as have a bearing upon the subject, as far as they have been ascertained.

This man, a native German, was aged 35 years—was about four feet high—had a very large head—body of medium length and size, but chest large in proportion to the remainder, being prominent and expansive in front, and increased backwards by posterior curvature of the spine, while the lateral diameters were also considerable—lower extremities, thighs slightly, but permanently, flexed upon the body, probably in consequence of having been always a great-deal in the sitting posture, legs permanently bent almost at right angles upon the thighs, which was probably from a congenital contraction of the flexor muscles of the thigh, as he had been so from

*This paper was read before the Louisville District Medical Association, at its June monthly meeting, in behalf of the committee on Post-mortem Examinations, Morbid Anatomy, and Pathology.

birth: the lower extremities were also considerably atrophied, probably from non-use. His sister, who accompanied him at the time of his admittance, stated that he had been idiotic, had curved spine, and flexed lower extremities, from birth—that he had never walked, and it had always been necessary to carry him about; but that he had enjoyed rather robust health. From the time of his admittance, he remained altogether in his bed, sometimes in the sitting but mostly in the recumbent posture--was observed to be an enormous eater—that his bowels were habitually constipated, there being usually an interval of from eight to fourteen days between the time of his having an evacuation, which always consisted of an immense bulk of hardened fecal matter—but that his general health remained good, having only an occasional attack of dyspnœa, which was *slight* in its character, of from half an hour to three quarters in duration, and brought on by mental excitement, such as being irritated by his fellow inmates, but more frequently by rising suddenly in bed. The attention of the visiting physician was never called to him, and the resident students never made any examination in reference to the action of the heart. He never complained of pain in the thorax or abdomen, until he was attacked with dysentery, when there was only the pain that is usually attendant upon that disease, which was prevailing in the house at the time, and of which he died. It is deemed unnecessary, by your committee, to detail the facts connected with his last illness, as they relate only to the dysentery under which he labored, and have no bearing upon the lesions to which we would call your attention.

The examination was made twenty-nine hours after death. Upon laying open the abdomen to examine the bowels in reference to the disease of which he died, the stomach was observed to be out of its usual place; or rather, there was only a small portion of its pyloric extremity visible. Tracing it from this, we found it empty, and that almost the whole organ, which was of maximum size, had passed through a large opening in the diaphragm into the cavity of the thorax. The opening consisted in an enlargement of the natural foramen for the transmission of the œsophagus, was circular in shape, fully two inches and a half in diameter, with its edges about a third of an inch thick. The peritoneum, as it covers the diaphragm, was reflected through the opening around its whole edge, passed vertically about four inches into the cavity of the thorax, thus forming a hollow cylinder of that length, and two inches and a half in diameter; and having reached that height, the membrane concentred upon the œsophagus, which was placed in the axis of the cylinder, and thence passed down it upon the stomach, and on to the other viscera. Thus was formed a *cul-de-sac*, in which the stomach was lying; but we should remark, however, that it was not perfectly cylindrical, for that it was enlarged above so as to make it somewhat balloon shaped. The peritoneum forming the sac, was thickened to about double of what it usually is. We found no lesion of any of the viscera of the abdomen, having any connexion with that of the diaphragm. Upon opening the thorax, the left cavity was observed to be somewhat smaller than the right, in consequence of the protruding stomach, which was found lying in the posterior mediastinum, trespassing slightly upon this cavity; but notwithstanding, it was sufficiently capacious to allow the natural function of the lung, which was in a normal condition. The right lung was adherent throughout almost its whole surface to the contiguous parts by old adhesions, and so firmly to the diaphragm as to require a very careful dissection to effect its separation. The stomach, (as has been observed,) with its containing peritoneal cul-de-sac, was lying in the posterior mediastinum, in the natural position of the œsophagus, inclining therefore to the left side of the spine, and infringing more upon the left than the right pleural sac. There was no perforation of either of the pleuræ; and at the base of the protruding peritoneal sac, for near an inch above the edge of the diaphragm, the left pleura, as it commences ascending to form the mediastinum, was separated from the peritoneum by only a thin layer of cellular substance; but above this a greater amount of loose cellular texture was interposed between them. The protruding parts were not found to pass sufficiently high and to the left to offer any mechanical interference with the action of the heart, which was in a normal condition; but it will be recollected that we could gather no facts relative to the manner in which this organ discharged its duty.

Whether as mere coincidences, or as having a necessary connexion with those already described, we pretend not to say, but as occurring in like structures, your committee deem it right to mention briefly some lesions which were found in the head; and the condition of the spine to which the curvature was owing. In the first place, the bones of the head generally, but particularly those forming the vault of the cranium, were in a state of manifest hypertrophy, being in some places well nigh half an inch thick. On their inner surface, (especially the parietal and frontal bones.) were found a considerable number of exostoses, in the form of conical projections, about the fourth of an inch high, and the third of an inch in diameter at the base. In employing the term exostosis, we intend it to signify "a bony excrescence, similar in its structure to the osseous tissue in its normal condition," as it is defined by Professor Gross in his work on Pathological Anatomy. The dura-mater adhered with unusual firmness to the skull, but particularly to these projections. On the inner surface of the dura-mater were found a number of osseous deposits. These existed most abundantly in the falx cerebrum, along the superior longitudinal sinus; where they varied in size from masses of an oblong shape, an inch long and a half an inch wide, to mere points. There were some of them scattered throughout the whole of the falx major and tentorium; and wherever found consisted of very hard, white osseous masses, very rough on their free or cerebral surface, but smooth on that by which they adhered to the dura-mater. The distortion of the spine consisted in a posterior curvature, which was somewhat gradual between the first dorsal and last lumbar vertebræ, being greatest however at the middle of the dorsal, where the deviation from the natural position was about two inches and a half. There was also an observable curvature to the right side. The bony structure of the column seemed to be in a perfectly healthy condition; but the intervertebral substance, in all the spaces included within the curvature, was deficient in its anterior part, and from the first to the eighth dorsal vertebra, the absorption was so complete as to bring the anterior edges of the vertebræ immediately into contact. This absorption or atrophy of the intervertebral substance, may have happened in one of two ways: either as an original, or congenital, mal-formation; or by atrophy of the erector muscles of the back, by which the parts in front of the spine would preponderate and cause the anterior portion of the intervertebral substance to be unduly pressed, and absorption result as a consequence. The former we think most probably true, as it was stated, by his sister, that the curvature existed from birth.

Your committee having thus given an account of this individual case, as briefly as was compatible with perspicuity, think that the infrequency with which this subject is brought before the profession, added to the fact, that it is unquestionably one of no little practical importance, from the incorrect diagnosis which is almost invariably made, and the equally erroneous, if not injurious, practice that is employed, when cases of the disease occur, we say, from such considerations, your committee think that it will not be altogether inappropriate to add some general remarks on the nature, varieties, causes, and symptoms of the disease. And in doing so, they trust that they will not be considered to trespass upon the time and patience of the society.

The disease may be defined to be, a protrusion of one or more of the abdominal viscera through a natural opening, deficiency, or wound, of the diaphragm, into the cavity of the thorax.

It may occur at any period, from fœtal existence to the termination of life; but from the cases mentioned and referred to by the authors whom we have consulted, and such as we have been able to collect from a large number of periodicals, we are induced to believe that the lesion, by which protrusion takes place, is congenital in by far the greatest number of instances. This probably happens from the inverted position which the fœtus generally assumes, whereby the pressure of the viscera of the abdomen may cause a protrusion, either by dilating some one of the natural openings of the diaphragm; or by causing a separation of its muscular or tendinous fibres, at some part where an uncommon laxity of them may exist; or, possibly, from some accidental pressure upon a particular part, by which the process of nutrition is prevented, and an unnatural opening thus produced. We merely throw these out as suggestions; being fully aware that the determining causes of malformations are enveloped in great mystery. The existence of even a large protrusion does not materially interfere with the well-being of the fœtus; for the function of respiration being not yet called into requisition, the mere mal-position of the organs could not affect its health and general development. In regard to the comparative frequency of its occurrence at different periods of *extra-uterine* life, we have not facts sufficient to justify an opinion, and authors are silent upon the subject. Spontaneous rupture is not apt to take place; because, from position, the forces which usually produce rupture in other parts of the abdomen cannot act with any effect upon the diaphragm. Indeed, during the period of existence alluded to, the disease arises almost invariably from wounds, and they may of course occur with equal facility at all periods, so far as the structures themselves are concerned.

Protrusion happens more frequently on the left than the right side of the diaphragm. This arises from the fact, that a kind of protection is given to the diaphragm by the liver, which lies in contact with the most of its right half, and which itself, from the extent of its surface, is not apt to protrude through any natural or unnatural opening which may exist. Instances however have occurred in which it has taken place on the right side. Sir Astley Cooper* refers to two

*On Hernia.

cases; in one, some of the "abdominal viscera had passed into the right side of the thorax;" and in the other, "the right extremity of the stomach, and beginning of the duodenum, a part of the omentum, and the arch of the colon" had passed into the right side, through an opening situated a little distance from the right side of the ensiform cartilage. Mr. Lawrence† also refers to cases in which the protrusion occurred on the right side. In one, a child that died shortly after birth, the whole liver had passed into the right side of the chest; in the other, "part of the colon, and nearly the whole of the omentum," had passed into the right cavity through an opening near the gall-bladder.

According to the division of Sir Astley Cooper, which is founded in nature, and we deem the best that can be made, there are three causes or varieties of this disease, as we have already intimated in the definition which we gave it, viz: first, where it results from malformation of the diaphragm, such as a preternatural opening; secondly, where protrusion takes place through one of the natural foramina, enlarged from whatever cause; thirdly, from wounds.

*First of malformations.* Not taking into the account those protrusions which take place from wounds, this variety is by far the most common. Why this occurs more frequently than protrusion through the natural foramina, it is difficult to say; for the reason that the determining cause of these lesions is wholly beyond our reach. We have already said that both of these lesions are much more apt to take place on the left than right side, and we assigned the most probable reason for it; and in regard to that under consideration, we may safely say that it occurs more frequently in the muscular than in the tendinous portion of the diaphragm. This may arise from a greater tendency to laxity, or to use better language, *atrophy*, of the muscular than tendinous structure; by which the same mechanical pressure, of the superincumbent viscera in the inverted position of the fœtus, would effect a hernia in the muscular, when it would not in the tendinous portion. For

†Treatise on Hernia. London, 1838.

it will be recollected that these lesions are of almost infinitely greater frequency in fœtal existence than afterwards; and in regard to the greater tendency to *atrophy* of the muscular than tendinous structure at this period, we are strongly supported by the analogy of its greater frequency in extra-uterine life.

In the variety of the disease which we are now considering, the unnatural opening may have a sac formed by the peritoneum and pleura; or these membranes may terminate at the margin of the opening by running into each other, and thus give it a rounded and smooth edge. The comparative frequency of these states is decidedly in favor of that in which the sac does not exist.

When an opening of this kind exists, whether supplied with a sac or not, a temporary protrusion may take place, of a greater or less amount of the abdominal viscera, according to the size of the opening, and the force causing the protrusion, bringing on such symptoms as will be mentioned in a subsequent part of this paper; and after a greater or less time, according to circumstances, the parts may return to their natural cavity, with a subsidence of all the symptoms. Or on the other hand, inflammatory action may be set up, and, if gangrene do not result, adhesions will be produced, and the parts retained in their unnatural position.

In illustrations of these several propositions, we might adduce a considerable number of cases, but we must content ourselves with a brief allusion to a few.

In the 9th volume of the Transactions of the Royal Society of London, Fothergill relates the case of a child that suffered from laborious respiration and disordered bowels up to the time that it was ten months old, when it was weaned; in six days after this it was seized with violent vomiting, which terminated fatally in twenty-four hours. Upon examination, a large portion of the stomach, the cœcum, a large portion of the ileum, and a portion of colon, were found in the left side of the chest; having passed through an opening which extended from the sternum and cartilages in front, down to the tendinous centre of the diaphragm. We have already alluded to a case given by Sir Astley Cooper, in which some of the abdominal viscera had passed into right side of the chest, through an opening a little to the right of the ensiform cartilage. We must again refer to it, as it presents the interesting feature of a sac formed by the pleura and peritoneum, and which contained the protruding parts. In the 19th vol. of the Med. Chirurg. Rev. is an abstract of a case, by Cruvielhier, in the person of a female aged 75 years, rachitic, and for several years subject to temporary colics. She died labouring under the ordinary symptoms of hernia, attended with constant vomiting. The stomach, large intestine, and some portions of small intestine were found contained in "a sac," in the left side of the thorax. Another case is detailed at some length by Sir Astley Cooper, of a woman aged twenty-eight years, who had from childhood suffered pain in the left side, slight cough, and difficulty of respiration; and died laboring under symptoms of strangulated hernia. An opening was found in the muscular portion of the diaphragm, three inches from the œsophagus, circular in shape, two and a half inches in diameter, with the peritoneum terminating abruptly at the edge, which was smooth and somewhat thickened. The protruding parts (the arch of the colon, and nearly the whole of the omentum) were in a state of incipient gangrene; and the omentum adhered firmly to the edge of the aperture. In the Lancet for April, 1832, Mr. Edwards relates a very interesting case, of a "delicate female, of fine complexion, and exquisite symmetry, who had from infancy enjoyed imperfect health. She was subject to frequent attacks of dyspnœa attended with acute pain in the region of the heart. These came on suddenly, without any apparent cause, and went off as unaccountably." She married at the age of 22, and soon after became pregnant, when the attacks of dyspnœa became more frequent and of longer continuance; but by great care, she went on to full term. At this period, soon after feeling what she supposed to be labour pains, she had an attack of dyspnœa, which became much worse than usual, and after suffering considerably from vomiting, purging, and great prostration, she died in nine hours from the commencement of the attack. On examination, the arch of the colon, the omentum, and pyloric half of the stomach, were found almost filling the left pleura, compressing the lung against the mediastinum. The protrusion had taken place through an opening a little anterior to the cordiform tendon, the peritoneum and pleura terminating at its margin, which was bounded by a tendinous ring. There was, of course, no investing sac to the protruding parts, which were adherent to different points of the pleura, but not to the edge of the opening. They were also gangrenous. No lesion of the heart.

Besides these, as illustrating the same points, we might quote a case from the 27th vol. of the Med. Chirurg. Rev.—one from the 26th vol. of the Edinburg Med. and Surg. Journal—and one from the 14th vol. of the Philadelphia Med. Journal, together with several from Sir Astley Cooper and Mr. Lawrence. But those which we have noticed are sufficient for our purpose; and circumstances impose upon us the propriety of perhaps even greater brevity than we have used.

We next come to speak of hernia, as it occurs at *the natural apertures.* This is not only comparatively but absolutely of rare occurrence. And this fact gives strength to the suggestion which we made when speaking of malformations of the muscular portion, viz: that *atrophy* has not a little to do in the matter. For leaving this out of the question, we conceive that, in the fœtus, hernia would be much more likely to occur at the natural apertures, than by a mechanical separation of the muscular fibres, by superincumbent pressure, as some have supposed. Why this species of hernia occurs so rarely notwithstanding the favorable position which the fœtus assumes, we are not able to say; but Nature probably gives to these apertures a protection which we do not well understand. That it does not occur more frequently in extra-uterine life, is sufficiently plain; for the forces tending to produce rupture in other regions of the abdomen, here have to operate against the gravity of the parts to be thrust out. To the fact itself, of its rare occurrence, we have the united testimony of the great barrenness of periodicals in such cases, and the opinions of the best authors upon the subject. Indeed in a very large number of periodicals which we have searched, we have not been able to find a single case of the kind related. Sir Astley Cooper and Mr. Lawrence, who have written the most extensive and systematic treatises on hernia, and who are the only authors, within our reach, who treat at all of *diaphragmatic hernia*, concur in the opinion that this variety is very rare. The former says that he never met with an example of it; and refers to but two cases, in Morgagni. In one of these, "part of the colon, a large portion of the omentum and pancreas" had passed through the opening for the transmission of the intercostal nerve. In the other, the duodenum, jejunum, part of the ileum, and omentum, had passed into the thorax through the opening which transmits the œsophagus. This occurred in a young man, who was suddenly attacked with severe pain in the region of the stomach, and vomiting of a great quantity of blackish matter, but whose previous history is not mentioned. The reference is also wanting in a statement as to whether the parts were contained in a sac; but from their great magnitude, compressing, as he says, the heart and lungs into a narrow compass, we suppose that no sac existed. The latter author (Mr. Lawrence) is silent in regard to his own experience, and we therefore think it fair to presume that he never met with an instance of it. He refers to but one case, in which his own mind was satisfied upon the subject; in that, the stomach and part of the omentum had passed into the chest, through the œsophageal opening. So far then as our research has extended, we have not been able to find a case on record precisely parallel to the one described in the first part of this communication.

Lastly, hernia proceeding from *wounds of the diaphragm*, will occupy our attention. In considering the comparative frequency of the varieties of the diaphragmatic hernia, we have not taken this last into the account; for as the same circumstances could not influence their production, we did not deem such a comparison entirely legitimate. In regard to the absolute frequency of the occurrence of this last, it is probably greater than would at first be supposed, from considering the position of the diaphragm in the body. Such wounds *might* happen in various ways, but they *have* happened most frequently from cutting weapons, such as swords and knives, and the fracture of ribs. Of course we say nothing of the comparative frequency of their occurrence in different regions of the diaphragm; for happening as they do, in every instance, from external violence, they may take place with equal facility in any part of it. But an abstract of some cases which we are enabled to present, will give a better idea of the nature and varieties of this accident, than any general remarks of our own.

In the "Revue Medicale" for July, 1823, Cloquet relates a case in which a man had his thorax compressed from before backwards by two carriage wheels. He died in thirty-six hours after labouring under great pain in the chest, difficulty of respiration, and accelerated and intermittent pulse. On examination, the soft parts of the walls of the thorax were not lacerated, but several ribs were fractured, and there was found a rupture of the diaphragm on the left side, extending towards the centre, through which the whole of the stomach, and most of the colon, had passed into the left side of the chest, greatly compressing the heart and lungs. In the 4th vol. of the Brit. and For. Med. Rev. is mentioned a case of a man who received an injury of the chest by falling from a great height, which brought on great pain near the sternum, dyspnœa, and respiration only on the right side—death following after a few days. The eight superior ribs of the left side were found fractured, and a laceration of the diaphragm, on the left side, to the extent of four or five inches, through which the stomach, half of the duodenum, spleen, transverse colon, and the edge of the left lobe of the liver, had passed into the left pleura. Sir Astley Cooper mentions a case which came within his own observation. It happened in the person of a man, who fell from a considerable height, and received the shock of the fall on his back against the edge of a pump. He had great pain, and difficulty of breathing, violent vomiting and hiccough, and died in about nineteen hours. Several of the ribs of the right side were found fractured, the extremity of one of which had penetrated the diaphragm, producing a laceration of some considerable extent, through which a portion of the ileum had protruded, and become so strangulated by the edge of the muscle as to be in a state of incipient gangrene. He also mentions another furnished him by a surgeon in the British navy. A man fell, from the height of six feet, on his left side; soon after had nausea, vomiting, restlessness, difficulty of respiration, feeble and scarcely perceptible pulse, and obstinately constipated bowels, all of which increased until the fifth day, when he died. There was found a rupture of the tendinous part of the diaphragm, of near three inches in diameter, through which the stomach, and part of the duodenum, had passed into the left side of the chest. The stomach was greatly distended, and occupied almost the whole of the left pleura, greatly compressing the lung and heart. Mr. Lawrence quotes a case from the 10th volume of London Med. Gazette, of a man who was wounded by a broad pointed knife, which entered the left side, "between the fifth and sixth ribs, and penetrated to a considerable depth." A small portion of the left lung protruded through the external wound, was excised, and he recovered. From this time he suffered spasms in the region of the stomach, but was able to follow his occupation of steward on a ship, for several years. He was at length attacked with severe pain in the abdomen, attended with vomiting and constipation of the bowels; stercoraceous vomiting came on, and death took place in fifteen days, "under circumstances clearly indicating internal strangulation," says the author. An opening was found in the tendinous part of the diaphragm, through which the whole of the omentum had passed into the left cavity of the chest, the portion lying in the orifice of the diaphragm being firmly tied to it by old adhesions, while a portion of the colon was embraced and "firmly constricted" by it.

We might, in addition, adduce several other cases, but think that these will suffice.

Having thus considered the three varieties of the disease, it only remains for us to say a word on the *symptoms* which usually attended it. Here we may abridge, by saying, that the ordinary symptoms of hernia, whether reducible or strangulated, attend protrusions of the abdominal viscera through the diaphragm. We have in addition, such a condition of things as would be supposed to result from the existence of a foreign body in the cavity of the thorax: such as pain, difficulty of respiration, and greater or less irregularity in the action of the heart. And notwithstanding Mr. Lawrence asserts that it is impossible to make out the diagnosis, yet we think that where an opening exists, by which some of the abdominal viscera may protrude temporarily, and return again to their natural position, producing all the symptoms of hernia, together with dyspnœa, &c. which subside again suddenly, and without any obvious cause, we say, these symptoms, together with the non-existence of any thoracic disease to which the dyspnœa could be attributed, would strongly point to the disease under consideration as the cause of the symptoms.

